# A Multi-Region SEIR Model Incorporating Inter-County Mobility and Time-Dependent Transmission Dynamics: Application to COVID-19 Disease Outbreak Data in North Carolina.

**DOI:** 10.1007/s11538-026-01601-x

**Published:** 2026-02-12

**Authors:** Samuel R. Thornton, Erin C. S. Acquesta, Patrick D. Finley, Mansoor A. Haider

**Affiliations:** 1 Department of Mathematics, North Carolina State University, Raleigh, NC United States; 2 Sandia National Laboratories, Albuquerque, NM United States

**Keywords:** COVID-19 modeling, Infectious disease spread, Spatial mobility, County-level data

## Abstract

Classical infectious disease compartmental models typically do not incorporate spatial heterogeneity or mobility. We develop a multi-region susceptible-exposed-infected-recovered (SEIR) model in which disease dynamics are coupled via inter-region mobility and the transmission rate is both region and time dependent. We calibrate the model using rolling averages of daily COVID-19 data in all 100 North Carolina counties. Mobility parameters are prescribed using daily inter-county commuter data. The number of transmission rate parameters is substantially reduced by hypothesizing that the dynamics correlate with county-level population density. Parameter estimation is carried out using several objective functions with error terms at different scales. An additive combination of least squares error at the county-level and the state-level, along with a quadratic transmission rate polynomial, yields the lowest overall error at both spatial scales. The calibrated model is used to simulate regional effects of perturbing disease transmission rates in adjacent counties and to illustrate effects of the state’s mobility infrastructure on disease dynamics and spread for a new disease outbreak.

## Introduction

Compartmental models are widely used for studying the temporal dynamics of infectious diseases. The mathematical representation of such models, as systems of ordinary differential equations (ODEs), facilitates their rapid development and application to data-driven problems. Many applications in epidemiology and public health require more spatial granularity than is typically incorporated into the infectious disease model. For example, in the United States many policymaking decisions are interdependent, based on data and forecasting at both the county-level and the state-level (Institute of Medicine (US) Committee on Assuring the Health of the Public in the 21st Century 2003). Models incorporating more local or regional dynamics can account for factors such as inter-county mobility patterns, differences in outbreak prevention or mitigation policies, and human behavior across the counties within a state. The significance and impact of models incorporating these features will be greatest when they are calibrated using spatiotemporal data at both the finer and coarser spatial scales.

In this study, we develop a multi-region compartmental model for infectious disease dynamics that also accounts for mobility among geographic subregions (e.g., counties within a state). This model is developed and calibrated in the context of the COVID-19 pandemic using data at both the state and county levels for an outbreak that occurred in 2021.

Spatially structured mechanistic compartmental models, in which a population is divided into subpopulations by spatial regions, are referred to in the literature as metapopulation or patch models; see Colizza and Vespignani (2008) for a detailed summary. Among models that were calibrated to epidemic data, Bürger et al (2016) developed a multi-region SEIR model, accounting for mobility between 15 regions in Chile. The model was calibrated using hospitalization data from the 2009 H1N1 influenza pandemic. The transmission rate, latent period, and recovery rate parameters were all prescribed. Mobility parameters were estimated from least square fitting to peak timing of hospitalizations in each region.

Balcan et al (2009a) developed the GLEaM (GLobal Epidemic and Mobility) metapopulation model, which involves a network of 3,362 subpopulations centered around major airports in 220 different countries. Stochastic processes govern mobility between subpopulations as well as disease transmission within each subpopulation (see Balcan et al (2010)). Balcan et al (2009b) used a maximum likelihood estimate of the reproduction number based on the arrival time of newly infected countries during the 2009 H1N1 influenza pandemic. The method involves Monte Carlo generation of the distribution of arrival times of the infection in 12 countries based on $10^6$ simulations with the GLEaM model. Zhou et al (2020) developed a county-level forecast model that combined spatial cellular automata (CA) with a temporal extended susceptible-antibody-infectious-removed (eSAIR) model. The CA links counties via a spatial connectivity function characterizing the intercounty mobility, geodistance, and air-distance via accessibility to nearby airports. Separate state-level eSAIR model parameters were estimated from state case data using a Markov Chain Monte Carlo (MCMC) approach. The state-fit models combined with the CA were then used to project the county-level COVID-19 prevalence of 3,109 counties in the continental United States.

Chen et al (2020) implemented a multi-region SEIR with explicit mobility between 50 U.S. states and the District of Columbia. Mobility parameters were obtained from cellphone data. Other model parameters were estimated using an ensemble Kalman filter from state-level cumulative infection data. Chang et al (2021) developed a mobility network-based metapopulation SEIR model to study the spread of COVID-19, separately, in 10 of the largest U.S. metropolitan areas. The network used hourly cellphone data to connect census block groups (CBG) to points of interest (POIs) in each metropolitan area. Separate SEIR systems are placed on each CBG; infections within a CBG are modeled via a binomial process while infections occurring due to travel to POIs are modeled through a Poisson process. After fixing other model parameters, three parameters were estimated from a least squares fit of daily new case data in each metropolitan area. These parameters included a constant (in time) transmission rate shared within CBGs, a scaling factor of the transmission rate shared at POIs, and the initial proportion of exposed individuals. Yang et al (2021a) fit a multi-region SEIR model over all U.S. counties, coupled with a mobility-dependent force of infection using U.S. census commuter data. County-dependent piecewise constant in time (over each week) transmission rates were estimated from least square regression of observed and estimated COVID-19 infections in each county-week from March to July 2020. The study focused on the effects of non-pharmaceutical interventions on the effective reproductive number of the disease. Gatto et al (2020) developed a multi-region extended SEIR model that included a network of 107 provinces in Italy connected by mobility from Census data. After fixing some parameters, a total of 12 model parameters were estimated using MCMC from province-level hospital and mortality data. The transmission rates were piecewise constant in time over three separate time periods corresponding to lockdown periods in Italy. Additionally, all parameters were chosen to have the same values in each province.

Hou et al (2021) developed a multi-region stochastic SEIR model with mobility to study an outbreak of COVID-19 in two separate counties in Wisconsin (USA). Their system of ODEs included stochastic differential equations for regional transmission rates. Regions within each county were determined by partitioning census tracts (roughly 150 in each county) into six regions in one county and eight regions in the other county. A weighted graph was used to represent daily mobility flows based on cellphone data in each tract. Mobility parameters in each of the (six or seven) regions were determined by aggregating weights in the graph representations. An ensemble Kalman filter method was used to predict state variables and estimate parameters of the model given cumulative infection data.

We build on the model in Hou et al (2021) by developing a multi-region SEIR model tailored to the 100 counties of North Carolina (USA). We incorporate mobility between counties in the state using daily commuter data available from the US Census Bureau (U.S. Census Bureau 2023). In Hou et al (2021), the exposed group infects the susceptible group, while the infectious group is assumed to isolate. However, similar to that classical SEIR model and its extensions (see, e.g., Perkins and España (2020)), we assume in our model that the exposed group is not yet infectious. Our model also allows the infectious group to be mobile, accounting for the case that infectious individuals do not isolate. We take the region-dependent transmission rate to be a polynomial in time, rather than a stochastic variable as in Hou et al (2021). In contrast to the aforementioned studies, we calibrate our model using both county-level and statewide infectious disease data. Our approach incorporates a reduced set of transmission rate parameters, based on an hypothesis that the associated dynamics correlate strongly with county-level population density.

We first introduce our multi-region SEIR model with mobility, incorporating a time-dependent transmission rate. Next, our approaches for sourcing and averaging daily infection and mobility data are outlined. We then present methods used for model calibration and parameter estimation, considering both county-level and statewide data during a disease outbreak. Estimated parameter values and model accuracy, relative to the data, are compared and evaluated for several data-driven models. Calibrated models are then used to simulate regional effects of a perturbed disease transmission rate in adjacent counties. We also simulate effects of a new disease outbreak, illustrating impacts of the state’s mobility infrastructure on disease dynamics and spread.

## Models and Methods

### Multi-region SEIR Model with Mobility

Consider a population divided into $n_{\textrm{reg}}$ regions (e.g., North Carolina counties comprise $n_{\textrm{reg}}= 100$ regions). Let $N_i$ be the total resident population of region *i* ($i = 1, \dots , n_{\textrm{reg}}$). We assume that the total resident population of each region remains constant over the time period considered. Define $S_i(t)$, $E_i(t)$, $I_i(t)$, $R_i(t)$ as the susceptible, exposed, infectious, and recovered populations, respectively, of region *i* ($i = 1, \dots , n_{\textrm{reg}}$).

**Fig. 1:**
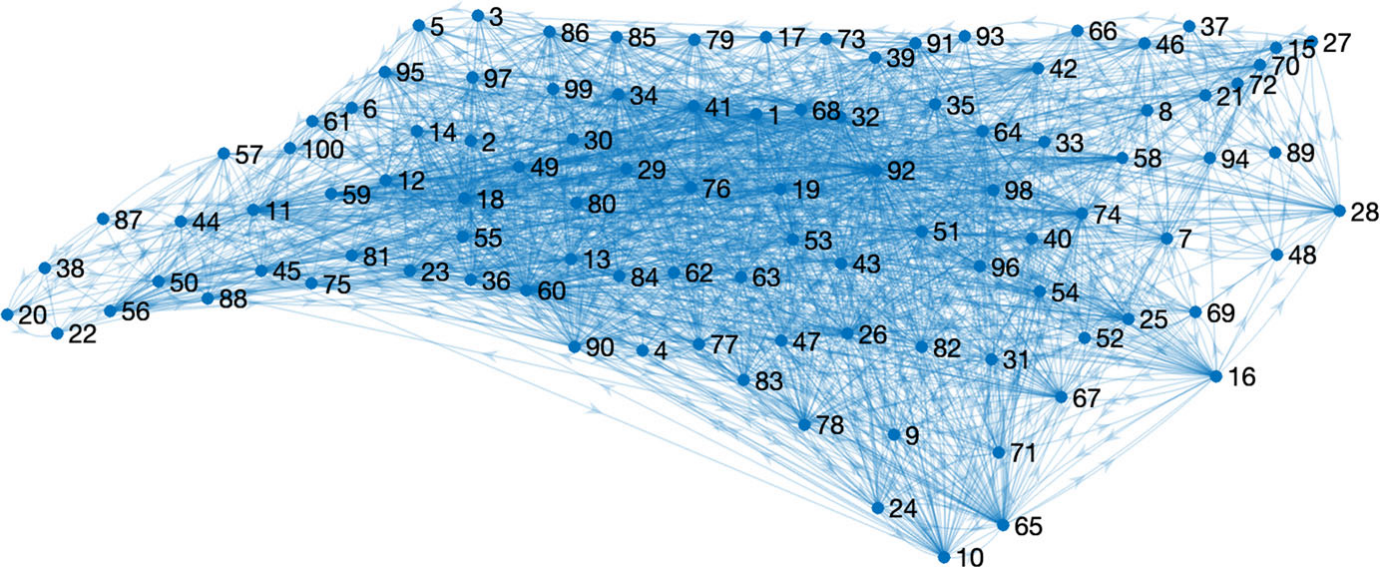
Graph of the 100 counties of North Carolina counties connected by daily commuters (U.S. Census Bureau 2023).

Let $f_{ij}$ denote the proportion of the resident population of region *i* that commutes daily to region *j* ($i,j = 1, \dots , n_{\textrm{reg}}$). Then define

1 $$\begin{aligned} {\tilde{N}}_i&:= N_i + \sum _{j\ne i} \Big [ f_{ji}N_j - f_{ij}N_i \Big ], \end{aligned}$$

2 $$\begin{aligned} {\tilde{S}}_i(t)&:= S_i(t) + \sum _{j\ne i} \Big [ f_{ji}S_j(t) - f_{ij}S_i(t) \Big ], \end{aligned}$$

3 $$\begin{aligned} {\tilde{I}}_i(t)&:= I_i(t) + \sum _{j\ne i} \Big [ f_{ji}I_j(t) - f_{ij}I_i(t) \Big ] , \end{aligned}$$

as the effective total population, effective susceptible population, and effective infectious population, respectively, of region *i* ($i = 1,\dots ,n_{\textrm{reg}}$). The regional mobility model in equations (1)-(3) assumes that daily commuters spend the working portion of each day in the region that they commute to. Their presence increases the effective susceptible and infectious populations of that region, thus also increasing the chances of infectious spread through interactions with the resident local population. We illustrate county-level commuter data, shown for the 100 counties in the state of North Carolina, in Figure 1.

Based on the assumptions and definitions above, we formulate a multi-region SEIR model with mobility as the following coupled system of ordinary differential equations. For each region $i = 1, \dots , n_{\textrm{reg}}$,

4 $$\begin{aligned} \frac{\textrm{d}S_i(t)}{\textrm{d}t}&= \frac{-\beta _i(t) {\tilde{I}}_i(t){\tilde{S}}_i(t)}{{\tilde{N}}_i}, \end{aligned}$$

5 $$\begin{aligned} \frac{\textrm{d}E_i(t)}{\textrm{d}t}&= \frac{\beta _i(t) {\tilde{I}}_i(t) {\tilde{S}}_i(t)}{{\tilde{N}}_i} - \rho E_i(t), \end{aligned}$$

6 $$\begin{aligned} \frac{\textrm{d}I_i(t)}{\textrm{d}t}&= \rho E_i(t) - \gamma I_i(t), \end{aligned}$$

7 $$\begin{aligned} \frac{\textrm{d}R_i(t)}{\textrm{d}t}&= \gamma I_i(t). \end{aligned}$$

Here, $\rho$ is the rate of progression from exposed to infectious and $\gamma$ is the recovery rate. These parameters are assumed to be globally constant, i.e., having the same value across all regions. Our model includes a time-dependent transmission rate $\beta _i(t)$ for each region. These transmission rates account for alterations in human behavior, public health mitigation measures, or other policy factors over the course of an outbreak, also capturing their variation from region to region. For our model, examples include policies and compliance related to masking, social distancing and quarantine, as well as supply-chain issues and vaccination status. The choice of transmission rate in our model provides a simple time-dependent mathematical form that captures multiple, diverse factors. The number of associated parameters can be greatly reduced through hypotheses that group together counties, based on demographic or other similarities (see Sec. 2.3).

In augmenting this model with initial conditions, we denote the daily new case counts on day $\tau$ in region *i* as $y_{i,\tau }^{\textrm{data}}$ ($i = 1,\dots ,n_{\textrm{reg}}$, $\tau = 0,\dots ,n_{\textrm{day}}-1$, where $n_{\textrm{day}}$ is the number of days of an outbreak). Assuming that $\tau =0$ corresponds to the initial day that data is available, our initial conditions are (for $i = 1,\dots ,n_{\textrm{reg}}$)

8 $$\begin{aligned} S_i(0) = N_i - y_{i,0}^{\textrm{data}}, \quad E_i(0) = 0, \quad I_i(0) = y_{i,0}^{\textrm{data}}, \quad R_i(0) = 0. \end{aligned}$$

For simplicity, we have assumed that the initial exposed and recovered populations are zero, consistent with modeling an outbreak associated with a new disease variant. We use the MATLAB routine ode113 to numerically integrate the ODE system (4–7).

### Data

*Infection Data.* We obtained daily cumulative North Carolina county-level COVID-19 case counts from the COVID-19 Data Repository at the Center for Systems Science and Engineering (CSSE) at Johns Hopkins University (Dong et al 2020). Using this data, we calculated raw county daily new case counts, $y^{\textrm{raw}}_{i,\tau }$ ($i = 1,\dots ,n_{\textrm{reg}}$, $\tau = 0, \dots , n_{\textrm{day}}$; $n_{\textrm{reg}}=100$ in the state of North Carolina).

Note that the raw daily new case counts exhibit some periodicity in that weekend days have zero new case counts; hence, Mondays have significantly higher new case counts compared to other days. To account for these factors, we take a centered 7-day rolling average of the data,

9 $$\begin{aligned} y^{\textrm{data}}_{i,\tau } = \frac{1}{7}\sum _{k = \tau -3}^{k = \tau +3} y^{\textrm{raw}}_{i,k}, \quad i = 1,\dots ,n_{\textrm{reg}}, \, \tau = 3, \dots , n_{\textrm{day}}-4. \end{aligned}$$

For days at the end points ($\tau = 0, 1, 2, n_{\textrm{day}}- 3, n_{\textrm{day}}- 2, n_{\textrm{day}}- 1$) the average is taken over only days in the centered 7-day sliding window.^1^

**Fig. 2:**
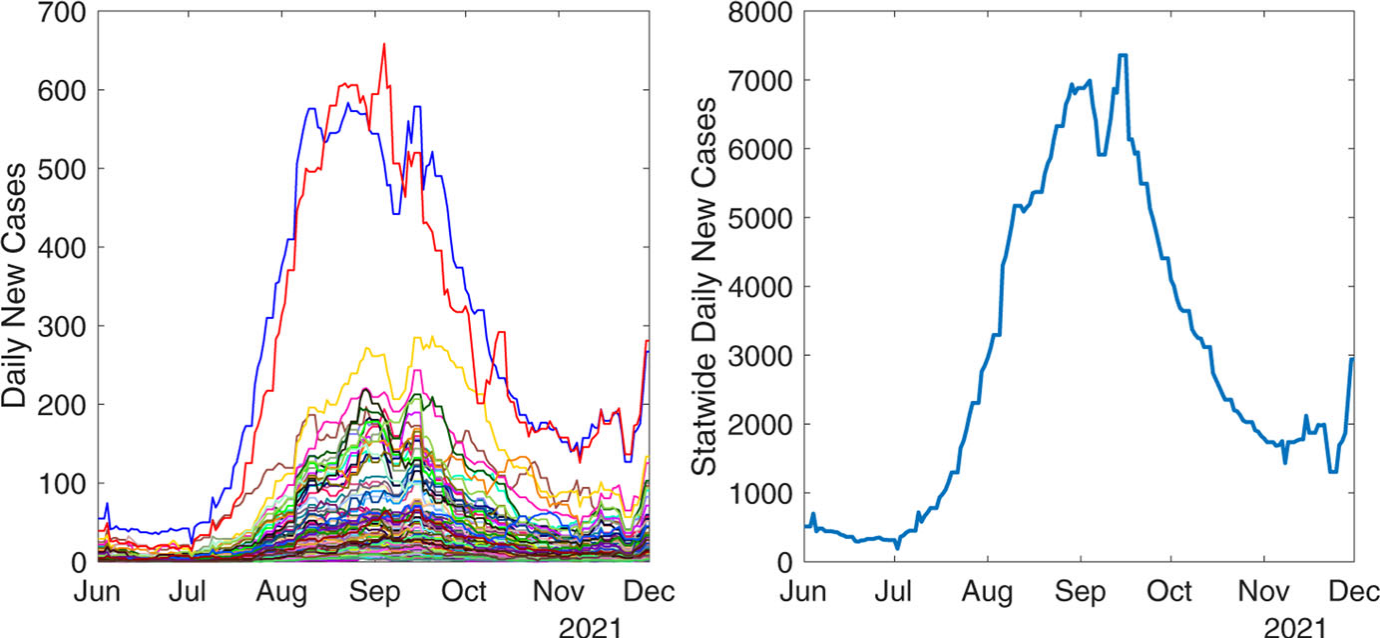
Centered 7-day rolling average of county-level NC COVID-19 data (9) where each curve corresponds to one county (left) and corresponding statewide sum (10) (right).

We plot $y^{\textrm{data}}_{i,\tau }$ and the corresponding statewide sum

10 $$\begin{aligned} Y_\tau ^{\textrm{data}} = \sum _{\ell =1}^{n_{\textrm{reg}}}y^{\textrm{data}}_{\ell ,\tau }, \end{aligned}$$

where $i = 1,\dots ,100$ and $\tau = 0, \dots , n_{\textrm{day}}-1$, in Figure 2 for an outbreak that occurred in North Carolina from June 1, 2021 to December 1, 2021. In section 3.1, we fit the data from June 15, 2021 to November 15, 2021 ($n_{\textrm{day}}= 154$) using our model.

**Fig. 3:**
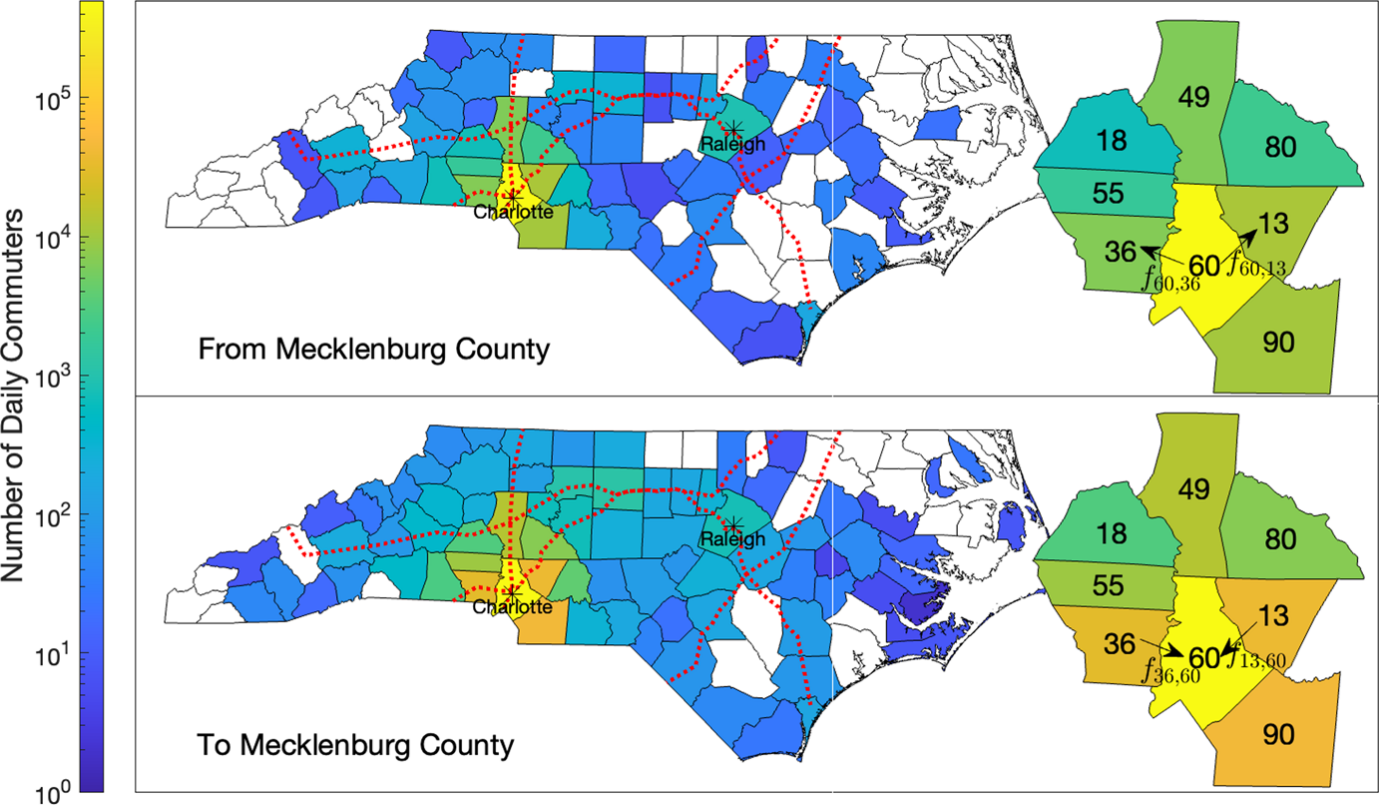
Choropleth plots of daily commuters to and from Mecklenburg County. The right choropleths show example indexing for the proportional commuter flow $f_{ij}$ from county *i* to *j* for Mecklenburg County (60) and surrounding counties. Interstates 40, 77, 85, and 95 are plotted with red dotted lines.

*Mobility Data.* We obtained daily commuting data for each county in North Carolina from the 2016-2020 American Commuter Survey (U.S. Census Bureau 2023). The data $r_{ij}$ is the number of residents of county *i* who commute daily to county *j* ($i,j = 1,\dots ,n_{\textrm{reg}}$). We determine quantities $f_{ij}$, the proportion of residents of county *i* who commute daily to county *j*, by dividing $r_{ij}$ by the population of county *i* ($i,j = 1,\dots ,n_{\textrm{reg}}$). Choropleth plots of number of daily commuters to and from Mecklenburg County—the most populous county in NC—and examples of indexing for $f_{ij}$ are shown in Figure 3.

### Parameter Estimation

As our model output, we calculate the daily new cases on day *t* in region *i* using the following formula,

11 $$\begin{aligned} y_{i}(t) = I_i(t) - I_i(t-1) + R_i(t) - R_i(t-1), \end{aligned}$$

where $i = 1,\dots ,n_{\textrm{reg}}, \, 0 \le t \le n_{\textrm{day}}- 1.$ Change in total infections is equal to daily new cases (flux in) minus new recoveries (flux out), which corresponds to (11). Note that cumulative cases on day *t* in region *i* can also be tracked by adding together $I_i(t)$ and $R_i(t)$.

For our initial objective function, we considered the sum of county-level squared errors, across all counties and over all days of the outbreak. That is,

12 $$\begin{aligned} {\mathcal {J}}_\textrm{co}(\boldsymbol{\theta }) = \sum _{\tau =0}^{n_{\textrm{day}}-1} \sum _{i=1}^{n_{\textrm{reg}}} \Big (y_i(\tau ; \boldsymbol{\theta }) - y_{i,\tau }^{\textrm{data}}\Big )^2, \end{aligned}$$

where $\boldsymbol{\theta }$ denotes the model parameters to be estimated. However, use of this objective function resulted in a significant underestimate of the statewide sum of daily new cases (see section 3.1). Thus, we chose to also incorporate the sum (over all days of the outbreak) of the statewide squared error,

13 $$\begin{aligned} {\mathcal {J}}_\textrm{st}(\boldsymbol{\theta }) = \sum _{\tau =0}^{n_{\textrm{day}}-1} \left( Y(\tau ; \boldsymbol{\theta }) - Y_\tau ^{\textrm{data}} \right) ^2, \end{aligned}$$

as an additional objective term, where $Y(t) = \sum _{i=1}^{n_{\textrm{reg}}} y_{i}(t)$. Consequently, the objective function used for parameter estimation is taken as the sum of a county-level objective term and a statewide objective term,

14 $$\begin{aligned} {\mathcal {J}}(\boldsymbol{\theta }) = {\mathcal {J}}_\textrm{co}(\boldsymbol{\theta }) + {\mathcal {J}}_\textrm{st}(\boldsymbol{\theta }). \end{aligned}$$

Recall that the transmission rate $\beta _i(t)$ is both time dependent and region dependent. We take $\beta _i(t)$ to be either a constant, linear, or quadratic polynomial in time. The same polynomial degree is used in each region; we denote this degree by *d*.

**Fig. 4:**
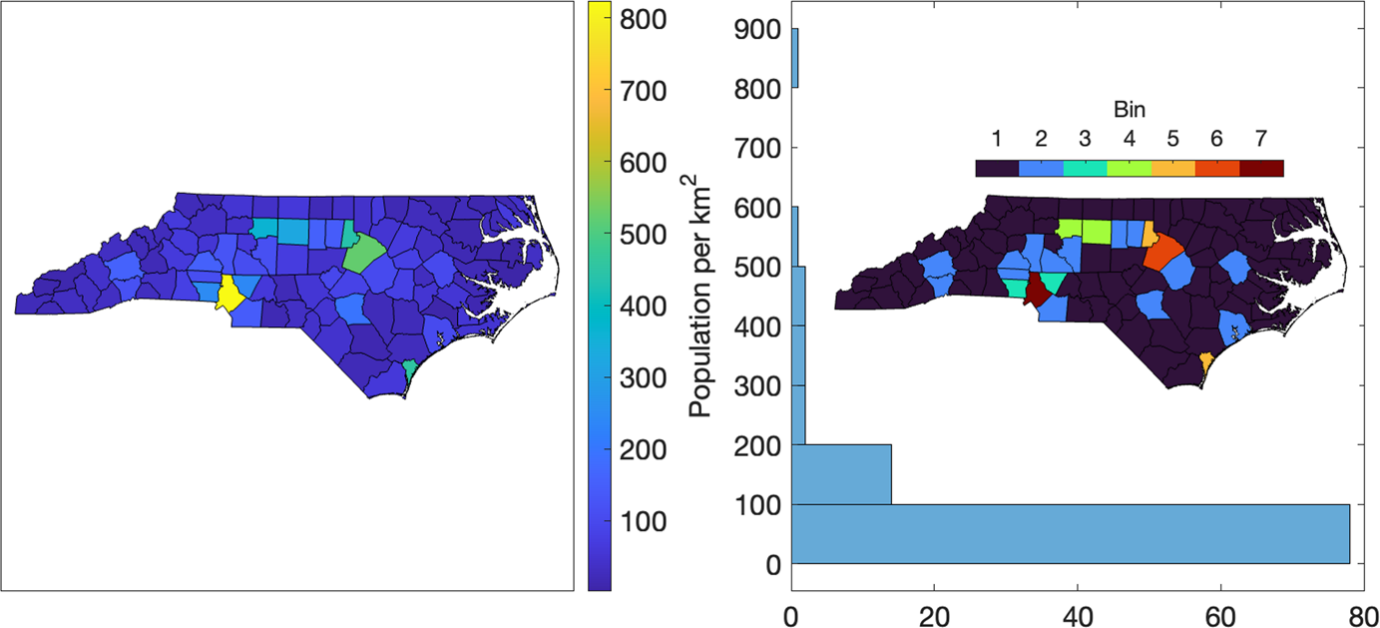
Choropleth plot and histogram of population density of NC counties (U.S. Census Bureau, Population Division 2024; U.S. Census Bureau 2021). The inset choropleth plot shows the spatial distribution of the 7 population density bins. Bins 1–7 correspond to lowest–highest population densities.

To reduce the overall number of parameters in the model, we assume that groups of counties with similar population densities exhibit similar transmission rates. For example, Ives and Bozzuto (2021) found that county population density is a significant predictor of rate of spread of COVID-19. Counties in each group, where groups are binned by population density, are assigned the same transmission rate polynomial. We denote transmission rate polynomial coefficients by $c_0^{(k)}, \dots , c_d^{(k)}$, for each bin $k=1,\dots ,n_{\textrm{bin}}$, where $n_{\textrm{bin}}$ is the number of nonempty bins. For North Carolina counties we made the choice $n_{\textrm{bin}}= 7$ (see Figure 4). Hence, the parameters to be estimated are

15 $$\begin{aligned} \boldsymbol{\theta }= \{\{c_0^{(k)}, \cdots , c_d^{(k)}\}_{k=1}^{n_{\textrm{bin}}}, \rho , \gamma \}, \end{aligned}$$

We use the MATLAB routine lsqnonlin to solve the non-linear least squares problem and obtain estimates for optimized parameter values,

16 $$\begin{aligned} \boldsymbol{\theta }^* = \arg \min _{\boldsymbol{\theta }} {\mathcal {J}}(\boldsymbol{\theta }), \end{aligned}$$

subject to the following constraints,

17 $$\begin{aligned} c_0^{(k)} \ge 0, \quad c_1^{(k)} \le 0, \quad c_2^{(k)} \ge 0, \quad (k=1,\dots ,n_{\textrm{bin}}) \end{aligned}$$

18 $$\begin{aligned} \tfrac{1}{5} \le \rho \le 1, \quad \tfrac{1}{14} \le \gamma \le \tfrac{1}{2}. \end{aligned}$$

Here, the units of $c_2^{(k)}$ and $c_1^{(k)}$ are $\textrm{days}^{-3}$ and $\textrm{days}^{-2}$, respectively; units of all other parameters are $\textrm{days}^{-1}$. Note that lsqnonlin uses a modified version of the fmincon ‘interior-point’ algorithm (interior-point-convex quadprog algorithm) to incorporate constraints. In (17), the range of the parameter $c_0$ is chosen to reflect its definition in a standard (constant infection rate) SEIR model, i.e., the product of the average number of contacts per person per time multiplied by the probability of disease transmission during a contact. In (17), the chosen range of $c_1$ ensures that a linear transmission rate is decreasing as time increases. For the quadratic transmission model, the chosen range of $c_2$ ensures that the associated parabola is concave up. The chosen range for $\rho$ corresponds to a latent period ($\rho ^{-1}$) between 1 and 5 days, while the range for $\gamma$ assumes a disease duration ($\gamma ^{-1}$) that is between 2 and 14 days. These ranges capture values of the latent period ($\sim$4 days) and the viral shedding duration ($\sim$10 days) reported in the literature for the Delta variant of COVID-19 in the Summer and Fall of 2021 (Grant et al 2022; Siedner et al 2021; Wang et al 2021).

To guarantee that the minimum value of the linear and quadratic transmission function values do not fall below zero we add the following additional constraints. For the linear transmission rate model we ensure $c_0^{(k)} + c_1^{(k)} n_{\textrm{day}}\ge 0$ ($k=1,\dots ,n_{\textrm{bin}}$). The analogous constraint for the quadratic transmission rate model is

$$\begin{aligned} c_2^{(k)} - \frac{(c_1^{(k)})^2}{4c_0^{(k)}} \ge 0, \quad k=1,\dots ,n_{\textrm{bin}}. \end{aligned}$$

The initial parameter estimates for multi-region SEIR model fits are chosen in the following manner. We first fit the data using the standard SEIR model with various configurations of initial transmission rate coefficients. We then record the optimal parameter values from the best fit and use these values for the initial parameter estimates in the multi-region SEIR fits. The parameters that we vary in the initial parameter sweep are the constant ($c_0$) and linear ($c_1$) coefficients of the transmission rate polynomial. We vary $c_0$ from 0.2 to 0.5 in steps of 0.05 and we vary $c_1$ from $-5\times 10^{-3}$ to $-1\times 10^{-3}$ in steps of $1\times 10^{-3}$. The other initial parameter estimates are $c_2 = 1\times 10^{-5}$, $\rho = \tfrac{1}{2}$, and $\gamma = \tfrac{1}{10}$. The recorded optimal parameter values in the quadratic case, which we use as initial parameter estimates in the multi-region SEIR fits are

19 $$\begin{aligned} c_0 = 0.35, \quad c_1 = -4.8 \times 10^{-3}, \quad c_2 = 1.8 \times 10^{-5}, \end{aligned}$$

20 $$\begin{aligned} \rho = 0.2, \quad \gamma = 7.1 \times 10^{-2}. \end{aligned}$$

Similarly, the initial parameter values used in the linear transmission rate models are

21 $$\begin{aligned} c_0 = 0.64, \quad c_1 = -1.5 \times 10^{-3}, \end{aligned}$$

22 $$\begin{aligned} \rho = 1, \quad \gamma = 0.5. \end{aligned}$$

## Results

### Model Calibration to NC COVID-19 Data

**Table 1:** Root mean square errors (RMSE) and Akiake Information Criterion (AIC) in fitting the multi-region SEIR model with mobility to NC COVID-19 data between June 15, 2021 and November 15, 2021 (rows 4–15), including comparisons with a standard SEIR model (rows 1–3). Both global (column 3) and regional (column 4) transmission rate models are considered. Comparisons of constant, linear, and quadratic transmission rate models are also shown.

Model	Objective	Global $\beta (t)^{a}$	Regional $\beta _i(t)^{b}$	State	County	AIC
				RMSE	RMSE	
SEIR	${\mathcal {J}}_\textrm{st}$	Constant	-	3204	-	$2.49 \times 10^3$
	${\mathcal {J}}_\textrm{st}$	Linear	-	501	-	$1.92 \times 10^3$
	${\mathcal {J}}_\textrm{st}$	Quadratic	-	289	-	$1.75 \times 10^3$
Multi-region SEIR	${\mathcal {J}}_\textrm{co}$	Constant	-	3310	56	$1.24 \times 10^5$
	${\mathcal {J}}_\textrm{co}$	Linear	-	1537	31	$1.06 \times 10^5$
	${\mathcal {J}}_\textrm{co}$	Quadratic	-	1224	27	$1.02 \times 10^5$
	${\mathcal {J}}_\textrm{co}$	-	Constant	3247	56	$1.24 \times 10^5$
	${\mathcal {J}}_\textrm{co}$	-	Linear	914	24	$9.81 \times 10^4$
	${\mathcal {J}}_\textrm{co}$	-	Quadratic	768	21	$9.41 \times 10^4$
	${\mathcal {J}}$	Constant	-	3192	61	$1.79 \times 10^5$
	${\mathcal {J}}$	Linear	-	508	50	$1.32 \times 10^5$
	${\mathcal {J}}$	Quadratic	-	291	37	$1.19 \times 10^5$
	${\mathcal {J}}$	-	Constant	3180	57	$1.79 \times 10^5$
	${\mathcal {J}}$	-	Linear	295	32	$1.17 \times 10^5$
	${\mathcal {J}}$	-	Quadratic	277	24	$1.11 \times 10^5$

$a^{a}$ A single transmission rate polynomial $\beta (t)$ is used for all regions.

${a}^b$ Multiple (seven) transmission rate polynomials are used with counties in the same bin, by population density, sharing the same transmission rate polynomial

We estimate parameters in our multi-region SEIR model in 12 different ways. For comparison, we also estimate parameters in a standard SEIR model for the three different transmission rate polynomials (Table 1, rows 1–3). We use the root mean square error (RMSE) between model predictions (11) and the NC COVID-19 data between June 15, 2021 and November 15, 2021 (9)–(10) to quantify the quality of the fits (Table 1, rows 4–15). For the multi-region SEIR model, we compare results when parameters are estimated using the county-level objective function (12) and the combined objective function (14). In each case, we make comparisons between the constant, linear, and quadratic transmission rate models, for both regional and global (statewide) transmission rate models.

We also compute the Akiake Information Criterion (AIC) for each of the 12 fits using

23 $$\begin{aligned} \text {AIC} = n \log (J(\boldsymbol{\theta }^*)/n) + 2(k + 1), \end{aligned}$$

where *n* is the number of data points, $J(\boldsymbol{\theta }^*)$ is the residual sum of squares (i.e., the value of the optimized objective function), and *k* is the number of model parameters (Banks and Joyner 2017). We use the AIC to compare model fits (e.g., Yang et al 2021b); note that we only compare AIC values for model fits using the same objective function (i.e., ${\mathcal {J}}_\textrm{st}$, ${\mathcal {J}}_\textrm{co}$, or ${\mathcal {J}}$).

**Fig. 5:**
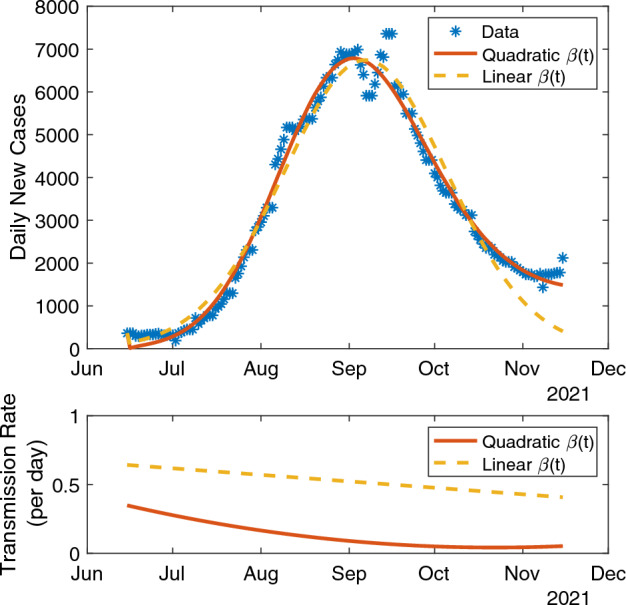
Fits of a standard SEIR model to the statewide data using a linear or quadratic transmission rate model $\beta (t)$.

For the standard SEIR model fit to statewide data, comparing the three transmission rate models (Table 1) reveals significantly better quality with the quadratic model (RMSE = 289) versus the linear model (RMSE = 501) and the constant model (RMSE = 3,204). The absolute difference between the AIC value for the quadratic model (AIC = $1.75 \times 10^3$) compared to the linear model (AIC = $1.92 \times 10^3$) and the constant model (AIC = $2.49 \times 10^3$) exceeds 100; hence the quadratic model is the best of the three SEIR models. The corresponding fits for model predictions of daily new cases to the data using the linear and quadratic transmission rate models are shown in Figure 5.

**Fig. 6:**
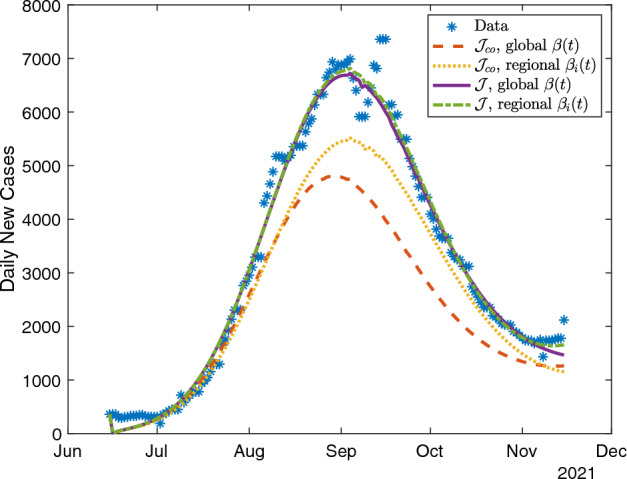
Fits of statewide data using the multi-region SEIR model with either the county-level objective function ${\mathcal {J}}_\textrm{co}$ (12) or combined objective function ${\mathcal {J}}$ (14) and either a single quadratic transmission rate polynomial $\beta (t)$ or multiple (seven) region-dependent quadratic transmission polynomials $\beta _i(t)$.

The quadratic transmission rate model also provides better quality fits for the multi-region SEIR model. The statewide RMSE values for the two objective functions ${\mathcal {J}}$ and ${\mathcal {J}}_\textrm{co}$ (respectively) using a regional transmission rate model in the quadratic case (RMSE = 768 and 277) are substantially lower than in the constant case (RMSE = 3,247 and 3,310), and lower than those in the linear case (RMSE = 914 and 295). We observe similar patterns for the global transmission rate model. Here, the quadratic case values increase (RMSE = 291 and 1,224) compared to the regional transmission rate model. We show fits for model predictions of daily new cases to the statewide data in the quadratic case in Figure 6.

Similar comparisons for the two objective functions ${\mathcal {J}}$ and ${\mathcal {J}}_\textrm{co}$ are made for the county-level RMSE. The quadratic case values (RMSE = 21 and 24) are significantly lower than in the constant case (RMSE = 56 and 57) for the regional transmission rate model. The analogous comparisons for the global transmission rate model also exhibit lower values in the quadratic case (RMSE = 27 and 37) as compared to the constant case (RMSE = 56 and 61).

The lowest AIC value for the ${\mathcal {J}}_\textrm{co}$ objective function is observed in the regional quadratic transmission model (AIC = $9.41 \times 10^4$), which exceeds 1000 in absolute difference compared to the other models for the ${\mathcal {J}}_\textrm{co}$ objective (AIC range from $9.81 \times 10^4$ to $1.24 \times 10^5)$. The lowest AIC value for the ${\mathcal {J}}$ objective is also observed in the regional quadratic transmission model (AIC = $1.11 \times 10^5$), which exceeds 1000 in absolute difference compared to the other models for the ${\mathcal {J}}$ objective function (AIC range from $1.17 \times 10^5$ to $1.79 \times 10^5$). This is evidence that the regional quadratic transmission rate models are the best of the models considered.

**Fig. 7:**
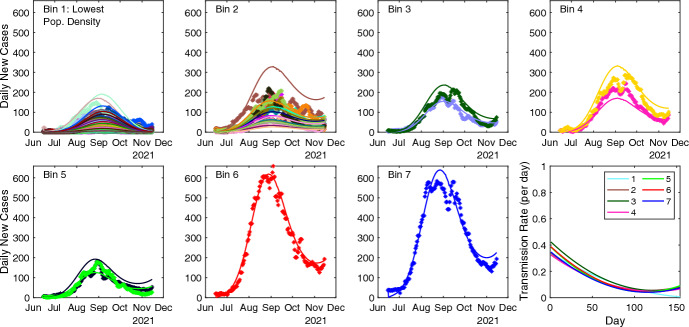
Multi-region SEIR fits of the county-level NC COVID-19 daily new case data with the state and county objective function ${\mathcal {J}}$. Region-dependent quadratic transmission rate polynomials determined by population density are plotted in last panel. The first seven panels correspond to population density bins, with the smallest population density bin in the first panel and the highest population density bin in the seventh panel.

The best overall combination of RMSE values at both the county and state levels is obtained using the multi-region SEIR model with a quadratic regional transmission rate when parameters are estimated using the combined objective function ${\mathcal {J}}$ (final row, Table 1). The best objective function values and the associated estimated parameters are provided in Table 2. We show the corresponding county-level fits, delineated by each of the seven bins, along with the seven (regional) quadratic transmission rate in Figure 7.

**Table 2:** Optimal objective function values and corresponding parameter values for the best fits of NC COVID-19 data using the standard SEIR model with quadratic transmission rate polynomial and the multi-region SEIR model with a global quadratic transmission rate or seven region-dependent quadratic transmission rate polynomials (rows 3,6,9,12, and 15 in Table 1).

Model	Objective		$c_0$	$c_1$	$c_2$	$\rho$	$\gamma$
SEIR	$\mathcal {J}_{st} = 1.37\times 10^7$		0.380	-0.00497	$1.90 \times 10^{-5}$	0.2	0.0891
multi-region SEIR	$\mathcal {J}_{\text {co}} = 1.14 \times 10^{7}$		0.35	-0.00499	$2.04 \times 10^{-5}$	0.2	0.0714
multi-region SEIR	$\mathcal {J}_{\text {co}} = 6.91 \times 10^6$	bin				0.202	0.0714
		1	0.301	-0.00310	$8.08\times 10^{-6}$		
		2	0.327	-0.00434	$1.69\times 10^{-5}$		
		3	0.419	-0.00573	$2.21\times 10^{-5}$		
		4	0.323	-0.00424	$1.61\times 10^{-5}$		
		5	0.387	-0.00609	$2.64\times 10^{-5}$		
		6	0.397	-0.00600	$2.53\times 10^{-5}$		
		7	0.352	-0.00533	$2.27\times 10^{-5}$		
multi-region SEIR	$\mathcal {J} = 3.42 \times 10^{7}$		0.35	-0.00476	$1.83 \times 10^{-5}$	0.2	0.0714
multi-region SEIR	$\mathcal {J} = 2.04 \times 10^7$	bin				0.202	0.0714
		1	0.338	-0.00380	$1.07\times 10^{-5}$		
		2	0.342	-0.00489	$2.09\times 10^{-5}$		
		3	0.429	-0.00605	$2.44\times 10^{-5}$		
		4	0.328	-0.00453	$1.83\times 10^{-5}$		
		5	0.389	-0.00619	$2.76\times 10^{-5}$		
		6	0.393	-0.00594	$2.51\times 10^{-5}$		
		7	0.352	-0.00537	$2.32\times 10^{-5}$		

**Fig. 8:**
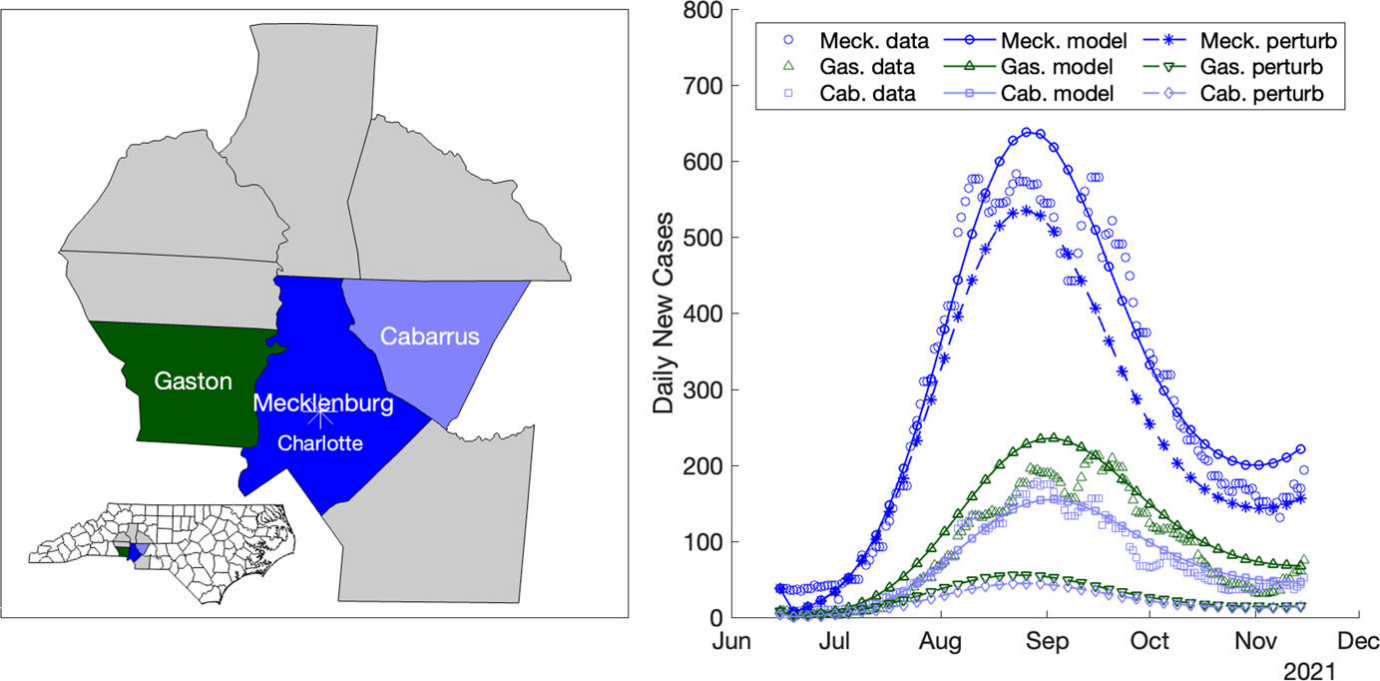
Simulation of a perturbation to calibrated NC COVID-19 multi-region SEIR model in which lower population density counties (Gaston Co. and Cabarrus Co.) adapt their quadratic transmission rate model to equal that of their neighboring high population density county (Mecklenburg Co.). Effects of the perturbation on the infection curves for the three counties is shown (right).

### Simulating statewide effects of a perturbed transmission rate

To investigate regional effects of the mobility dynamics inherent in our model, we consider a perturbation of the calibrated best-fit multi-region SEIR model outlined in the previous section (Table 1, last row). We simulate county-level effects, such as differences in behavior and/or policy, on regional infection dynamics. Specifically, we consider the dynamics between Mecklenburg Co., the most populous county in NC, and two of its bordering counties, Gaston Co. and Cabarrus Co. We perturb the quadratic transmission rates for Gaston Co. and Cabarrus Co. (bin 3 in Table 2) so that they are equal to the transmission rate of Mecklenburg Co. (bin 7 in Table 2, last row). We plot the data, the original calibrated model, and the perturbed model in Figure 8; note that the transmission rate for Mecklenburg Co. is not altered. We observe substantial reductions in infection levels and rates of infection in both Gaston Co. and Cabarrus Co. We also note significant downstream reductions in infection levels in Mecklenburg Co. (Figure 8, right panel), where model parameters were not perturbed.

**Fig. 9:**
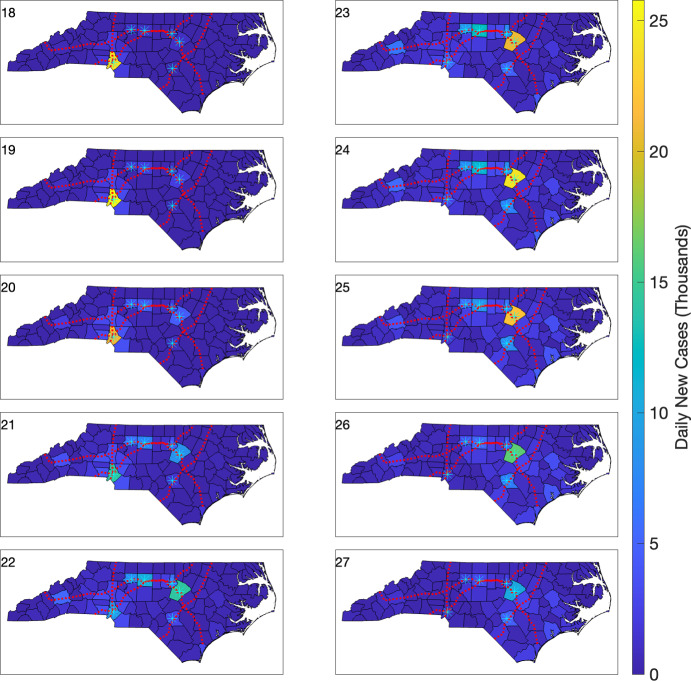
Choropleth plots of daily new cases during weeks 18–27 for the simulated outbreak model using a multi-region SEIR model. The infection is initiated with one infected individual in Mecklenburg Co. The six highest population cities (with populations above 200,000) are denoted by cyan asterisks. Interstates 40, 77, 85, and 95 are shown using red dotted lines.

### Simulating effects of mobility dynamics in a new outbreak

To investigate regional and statewide effects of mobility dynamics, we also simulate a new, isolated infection outbreak that starts in a single county. Since the data-driven features in such a simulation relate only to mobility, i.e., they do not involve parameter estimation from infection data, we use a global transmission rate model. Specifically, we prescribe the parameter values $\rho = \frac{1}{2} \, \textrm{days}^{-1}$, $\gamma = \frac{1}{7} \, \textrm{days}^{-1}$, and $\beta = 0.3 \, \textrm{days}^{-1}$.

**Fig. 10:**
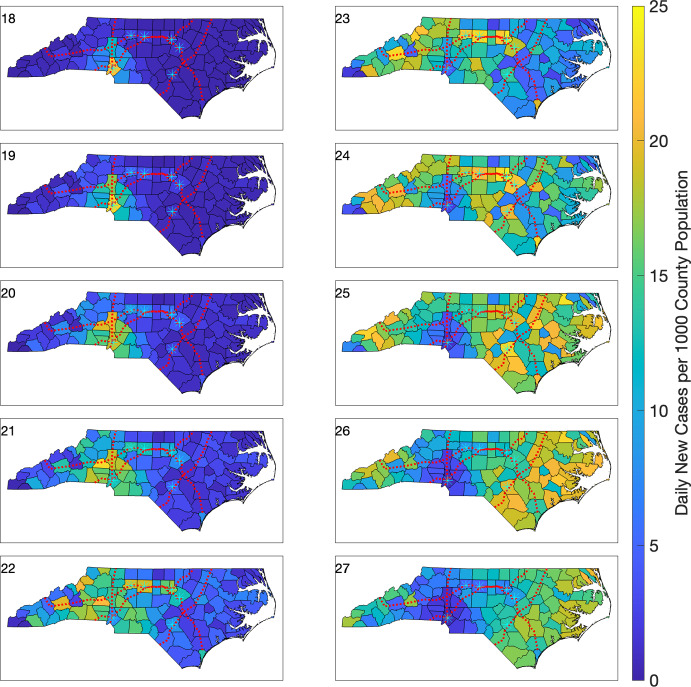
Choropleth plots of daily new cases per 1,000 individuals during weeks 18–27 for the simulated outbreak model using a multi-region SEIR model. The infection is initiated with one infected individual in Mecklenburg Co. The six highest population cities (with populations above 200,000) are denoted by cyan asterisks. Interstates 40, 77, 85, and 95 are shown using red dotted lines.

We initialize this model with one infected person in only Mecklenburg Co. and no exposed or recovered individuals in any county; the susceptible population is initialized as in (8). We show the resulting chloropleth plots between weeks 18 and 27 of the outbreak, for both daily new case counts (Figure 9), and for (scaled) daily new case counts per 1,000 individuals (Figure 10). The four major interstate highways that span NC, as well as several higher population NC cities, are also included in these plots.

We observe both spatial and temporal infection dynamics consistent with an initial peak in Mecklenburg Co. (Figure 9, week 18), followed by an infection rise and peak a few weeks later in Wake Co. (Figure 9, week 24); Wake Co. is NC’s second-most populous county. There is a similar lag in the time point at which infections subside in these two counties. More granular dynamics are apparent, in both space and time, when we employ scaled chloropleth plots (Figure 10). Here, we observe mobility effects that are geographically coincident with interstate highways that run through the county where the infection originated (Figure 10, weeks 18–20). A few weeks later, we observe the infection wave propagating to the north and east, in part, along two interstate highways (Figure 10, weeks 20–22). Interestingly, in week 23 an isolated hotspot emerges in the coastal county (New Hanover Co.) at the endpoint of an interstate highway that connects it with Wake Co. These dynamics illustrate a geographically nonlocal effect of incorporating mobility (e.g., commuters) in our model (Figure 10).

**Fig. 11:**
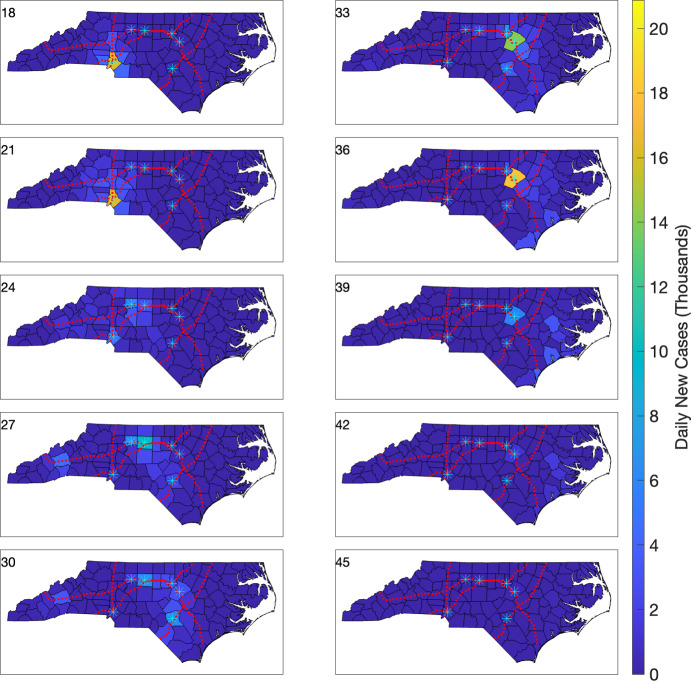
Choropleth plots of daily new cases during weeks 18–45 for the simulated outbreak model using a multi-region SEIR model with mobility determined by county adjacency. The infection is initiated with one infected individual in Mecklenburg Co. The six highest population cities (with populations above 200,000) are denoted by cyan asterisks. Interstates 40, 77, 85, and 95 are shown using red dotted lines.

**Fig. 12:**
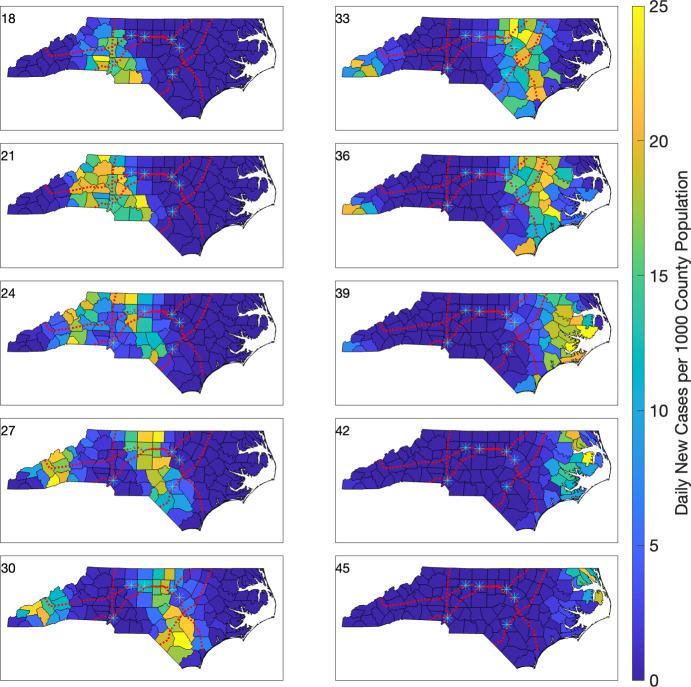
Choropleth plots of daily new cases per 1,000 individuals during weeks 18–45 for the simulated outbreak model using a multi-region SEIR model with mobility determined by county adjacency. The infection is initiated with one infected individual in Mecklenburg Co. The six highest population cities (with populations above 200,000) are denoted by cyan asterisks. Interstates 40, 77, 85, and 95 are shown using red dotted lines.

For a comparison, we consider the effect of replacing commuter data in mobility terms with less-informed values. We assume that for each county *i*, and for each bordering county *j*, one percent of the population of county *i* travels to (each) county *j*. That is, $f_{ij} = 0.01$ whenever counties *i* and *j* share a border, and $f_{ij} = 0$ otherwise. With such mobility terms, and the same prescribed parameter values and initial conditions outlined at the beginning of this section, we simulate another outbreak starting in Mecklenburg Co. We show the resulting cholorpleth plots between weeks 18 and 45 of the outbreak, for both daily new cases counts (Figure 11), and for (scaled) daily new case counts per 1,000 individuals (Figure 12).

Similar to the prior simulation, we observe both spatial and temporal infection dynamics consistent with an initial peak in Mecklenburg Co. (Figure 11, week 18), followed by an infection rise and peak a few weeks later in Wake Co. (Figure 11, week 36). When we employ the scaled choropleth plots (Figure 12), we observe that the propagation of the infection no longer reflects the geographic signature of the interstate highways. Instead, the infection first propagates to adjacent counties, taking about 45 weeks to spread to all counties of NC, compared to the roughly 25 weeks for the previous case.

Overall, our model captures coupled local, regional, and statewide spatiotemporal dynamics of infection initiation, spread, coalescence and recovery as the level of infections accelerates, peaks, and ultimately subsides across the state, and at different times over the course of the outbreak.

## Discussion and Conclusions

In this study we developed a data-driven multi-region SEIR model for county-level and state-level infectious disease dynamics. Some unique features of our model include a quadratic in time transmission rate that is also region-dependent, and the incorporation of mobility dynamics based on commuter data for the U.S. state of North Carolina. By hypothesizing that the disease dynamics correlate with county-level population density, we substantially reduced the number of transmission rate parameters in our model. The model was calibrated, and its parameters were estimated, using several approaches; an additive combination of county-level and statewide objective terms yielded the best result. Our model was also used to simulate effects of regional alterations in transmission rate in two counties neighboring the most populous NC county, simulating impacts on all three counties. Effects of a new disease outbreak in a single county were also simulated, demonstrating direct impacts of the state’s interstate highway network on disease dynamics and spread.

Among our calibrated models, the most accurate ones utilized a concave up quadratic disease transmission rate. This result is consistent with a combination of human behavior and mitigation factors. Specifically, these factors can lead to a rapid initial spread when there is poor awareness of a new strain, followed by a transmission rate that slows down as mitigation increases at both individual and collective levels. The small increase in transmission rate towards the end of the outbreak could be a combination of factors, one of which could be perception that the infection wave has subsided. Overall, the use of a quadratic transmission rate in a multi-region infectious disease model provides a richer model-form that implicitly captures such behavioral and policymaking factors, as compared to a linear or a constant transmission rate model.

Nevertheless, the multi-region infectious disease dynamics model presented in this study has several limitations. Since our underlying compartmental model is a SEIR model, it does not include a vaccinated population group (Perkins and España 2020). While vaccination increased during the time period used for model calibration, the transmission rate in our model also captures effects of vaccination status, yielding a simpler overall model. We also assumed that the initial exposed population was zero corresponding to the introduction of a new disease variant (the Delta variant); alternatively, the proportion of initial exposed could be estimated as in Chang et al (2021). Our model uses a deterministic relation to represent the transmission rate of the infections, whereas others have used a stochastic approach (Balcan et al 2009a; Chang et al 2021; Hou et al 2021). In addition, we estimate model parameters using a least squares fit, while others have used Bayesian approaches such as MCMC (Zhou et al 2020; Gatto et al 2020) or ensemble Kalman filter (Chen et al 2020; Hou et al 2021). Lastly, mobility in our model was incorporated using freely available commuter data between counties within a state. Using cellphone data (Chen et al 2020; Chang et al 2021; Hou et al 2021) in future studies would enable a more granular and possibly also a time-varying representation of mobility patterns and their effects on disease dynamics.

Lastly, our calibrated model assumes that transmission rates are the same for counties with similar population densities. Ives and Bozzuto (2021) found that population density is a significant predictor of county-level spread of COVID-19, but that population density alone explained only a modest fraction of the variation in transmission. Other factors—including population size, spatial location, and timing of outbreak—together with population density better capture county-level differences. We use population density as a proxy to simplify our model and reduce the number of estimated parameters. When applying our model to epidemiological forecasting, users should carefully evaluate the extent to which this assumption is valid for the particular application under consideration.

## Data Availability

Not applicable.

## Footnotes

1 Since no cases were reported on Labor Day, Monday September 6, 2021 and a significant increase in cases were reported on the Tuesday (Sept. 7) following Labor Day, after taking the 7-day rolling average, we observed a jump down in cases three days prior to Labor Day (Sept. 3) and a jump up in cases four days after Labor Day (Sept. 10). To resolve this discrepancy, we replaced the rolling average data on Sept. 3 with the mean of the rolling average data on Sept. 2 and 4. Similarly, we replaced the rolling average data on Sept. 10 with the mean of the rolling average on Sept. 9 and 11.

## Declarations

### Code availability

Code available upon request.
